# A cyclical deep learning based framework for simultaneous inverse and forward design of nanophotonic metasurfaces

**DOI:** 10.1038/s41598-020-76400-y

**Published:** 2020-11-10

**Authors:** Abhishek Mall, Abhijeet Patil, Amit Sethi, Anshuman Kumar

**Affiliations:** 1 Department of Physics, Indian Institute of Technology – Bombay, Mumbai, 400076 India; 2 Department of Electrical Engineering, Indian Institute of Technology – Bombay, Mumbai, 400076 India

**Keywords:** Electronics, photonics and device physics, Optical physics, Nanoscience and technology, Optics and photonics, Optical materials and structures

## Abstract

The conventional approach to nanophotonic metasurface design and optimization for a targeted electromagnetic response involves exploring large geometry and material spaces. This is a highly iterative process based on trial and error, which is computationally costly and time consuming. Moreover, the non-uniqueness of structural designs and high non-linearity between electromagnetic response and design makes this problem challenging. To model this unintuitive relationship between electromagnetic response and metasurface structural design as a probability distribution in the design space, we introduce a framework for inverse design of nanophotonic metasurfaces based on cyclical deep learning (DL). The proposed framework performs inverse design and optimization mechanism for the generation of meta-atoms and meta-molecules as metasurface units based on DL models and genetic algorithm. The framework includes consecutive DL models that emulate both numerical electromagnetic simulation and iterative processes of optimization, and generate optimized structural designs while simultaneously performing forward and inverse design tasks. A selection and evaluation of generated structural designs is performed by the genetic algorithm to construct a desired optical response and design space that mimics real world responses. Importantly, our cyclical generation framework also explores the space of new metasurface topologies. As an example application of the utility of our proposed architecture, we demonstrate the inverse design of gap-plasmon based half-wave plate metasurface for user-defined optical response. Our proposed technique can be easily generalized for designing nanophtonic metasurfaces for a wide range of targeted optical response.

## Introduction

The design and optimization of metasurfaces for unconventional functionalities has led to several new optical meta-devices for control over propagation of electromagnetic (EM) waves at sub-wavelength scale^1–5^. The constituent nanostructures of a metasurface are typically meta-atoms and meta-molecules that are designed for various light modulation applications^6,7^. Conventional design and optimization of metasurfaces components is iterative, based on trial and error, and reliant on physics-inspired approaches, e.g. numerical full-wave simulations using finite-difference time-domain (FDTD) or finite-element methods (FEM) for solving Maxwell’s EM equations. However, the development of optimized metasurfaces for various functionalities requires one to go beyond the limitations of physical intuition. Recently, deep learning (DL) has been used for inverse design and optimization of metasurface-based nanostructures for directed functionality^8–13^. With the use of multiple models based on DL, computational expense has been significantly reduced, and design and optimization processes have become highly efficient.

Unlike conventional design approaches, DL is a data-driven method. DL can learn highly non-linear functions mapping the inputs to the outputs in a training dataset by using deep artificial neural networks (NNs) with layer architectures that are amenable to training using convex optimization despite their depth. With a sufficient amount of training data and regularization techniques, the learned representation from DL can generalize to unseen datasets. Conventional fully connected (FC) NNs^14^ and convolutional neural networks (CNNs)^15^ have been used for nanophotonic metasurface design and optimization for the targeted optical response^16^. Most of these methods either encounter the limitation of optimizing a single candidate design or the requirement of a large dataset for the training process. NNs have also been used as a cascaded^17^ architecture with forward and inverse design networks for several targeted functionalities in nanophotonic metasurfaces. This NN architecture addresses the inverse design of nanophotonic metasurface as a regression problem, mapping optical response to structural design space. This approach however, forces the network to converge to one of the several solutions. These DL methods lack flexibility in designing nanophotonic structures because they usually limit the process of optimization to a predefined design of candidates and cannot generate new metasurface designs.

A few studies have tried to formulate the inverse design problem as modeling a conditional probability distribution of geometry/design for a given optical response^18,19^ using a conditional generative adversarial network (cGAN)^20^ and variational autoencoders (VAEs)^21^. Additionally, global optimization techniques such as genetic algorithms (GAs) have also proved useful for inverse nanophotonic design^22,23^. Nevertheless, generative approaches can often lead to structural designs with a deviating optical response and may require longer training and a larger dataset to generate highly efficient structural designs^9^. On the other hand, GAs face the issue of poor generalization capability to parameter space of different topologies^24^. Recently, simultaneous training of NNs performing forward and inverse mapping has shown promising results^25,26^. These approaches use generative models for inverse mapping and CNNs with multiple layers for the forward mapping. However, simultaneously training these NNs introduces problems in proper hyper-parameters selection due to co-dependence of both NNs and can result in poor convergence^25^.

In this paper, we propose a cyclical generation and discovery of metasurfaces for user-defined optical response, with no structural design restrictions during inverse design. Our framework consists of an efficiently trained deep generative model for inverse design and a simulation neural network (SNN) for forward design. The forward design is guided by a pseudo genetic algorithm (pGA). We use a conditional generative adversarial network (cGAN) as a generative model to model the probabilistic distribution of the design space. A trained cGAN generates new structural designs. In addition, the SNN is used to evaluate the authenticity of the structural designs generated by predicting the corresponding optical response. The pGA is applied to the optical responses to distinguish designs with minimal variance in their optical response compared to the desired optical response. It sorts generated structural designs to create a desired optical response and design space through selection and evaluation process. The proposed framework works cyclically to generate a desired optical response and design space that mitigates the need to collect data from traditional numerical simulation methods to train and analyze DL models. The optimized framework generates structural designs with 0.021 mean square error (MSE) and 0.968 cosine similarity, while also discovering new metasurface designs.

## Methodology

### Dataset preparation

**Figure 1:**
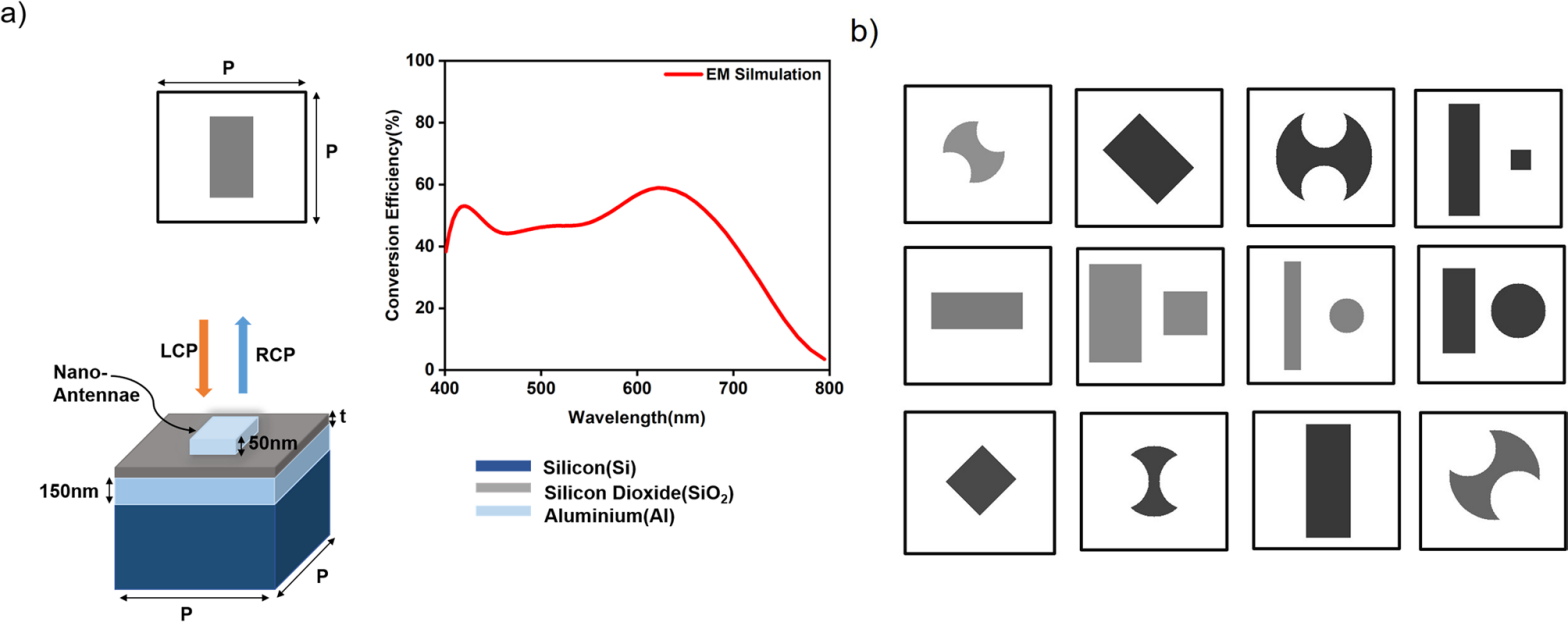
Illustration of gap-plasmon based HM. (**a**) A data sample composed of 2D cross sectional image of structural design of Al-nanoantennae and corresponding conversion efficiency as optical response. (**b**) Examples from structural design classes used for training of DL-NNs: cGAN and SNN. The pixel intensity of structural designs represents the thickness of the $\hbox {SiO}_2$ dielectric spacer.

For the training and evaluation of our method, we generated a dataset of 1500 Aluminum (Al)-nanoantennae samples as meta-atom and meta-molecule units^27^ of a periodic gap plasmon based half-wave plate metasurface (HM) with four classes of structural design (rectangle, double-arc, rectangle-circle pair, rectangle-square pair) of different dimensions. A typical sample from the dataset is shown in Fig. 1a, which is a pair of Al-nanoantennae structural design represented as a 2D-cross sectional image of $64 \times 64$ pixels and with corresponding conversion efficiency of an incident left circular polarization (LCP) to a right circular polarization (RCP) as optical response. The unit cell size of 230 nm x 230 nm with different structural design of antennae consists of Al-nanoantennae of thickness 50nm placed on a $\hbox {SiO}_2$ dielectric spacer over a 150nm thick Al-reflector on a silicon substrate (see Fig. 1a). The dielectric spacer thickness significantly affects the conversion efficiency, hence we encode the pixel intensity of structural designs to represent the dielectric spacer thickness (*t*) in range of 50-150 nm. The reflection conversion efficiency spectrum of each HM with 101 spectral points in wavelength range of 400 nm to 800 nm is obtained by FEM-based electromagnetic simulation software: COMSOL Multiphysics^28^.

A random selection of 75% of the 1500 samples was used for training and the remaining 25% was used for testing. As shown in Fig. 1b, the dataset with different structural designs of varying dimensions were used for the training of DL models. The DL models used solved metasurface design and optimization problem for a fixed unit cell size, periodicity, and wavelength range. Nonetheless, this approach to design and optimization is not limited to these assumptions and can be generalized by adding more design groups for nanoantennae with variable dimensions. Additionally, the proposed DL models and the cyclical generation framework can be used to incorporate additional geometrical or material parameters by encoding the information as pixel intensity in the image or concatenated at input node^12^.

### Configuration and training of DL models

We implemented the DL models using PyTorch library with hyper-parameters of the training process as mentioned in Table III in the Supplementary Information. In the proposed framework, the two DL models act as forward design and inverse design network.

#### Forward design network

**Figure 2:**
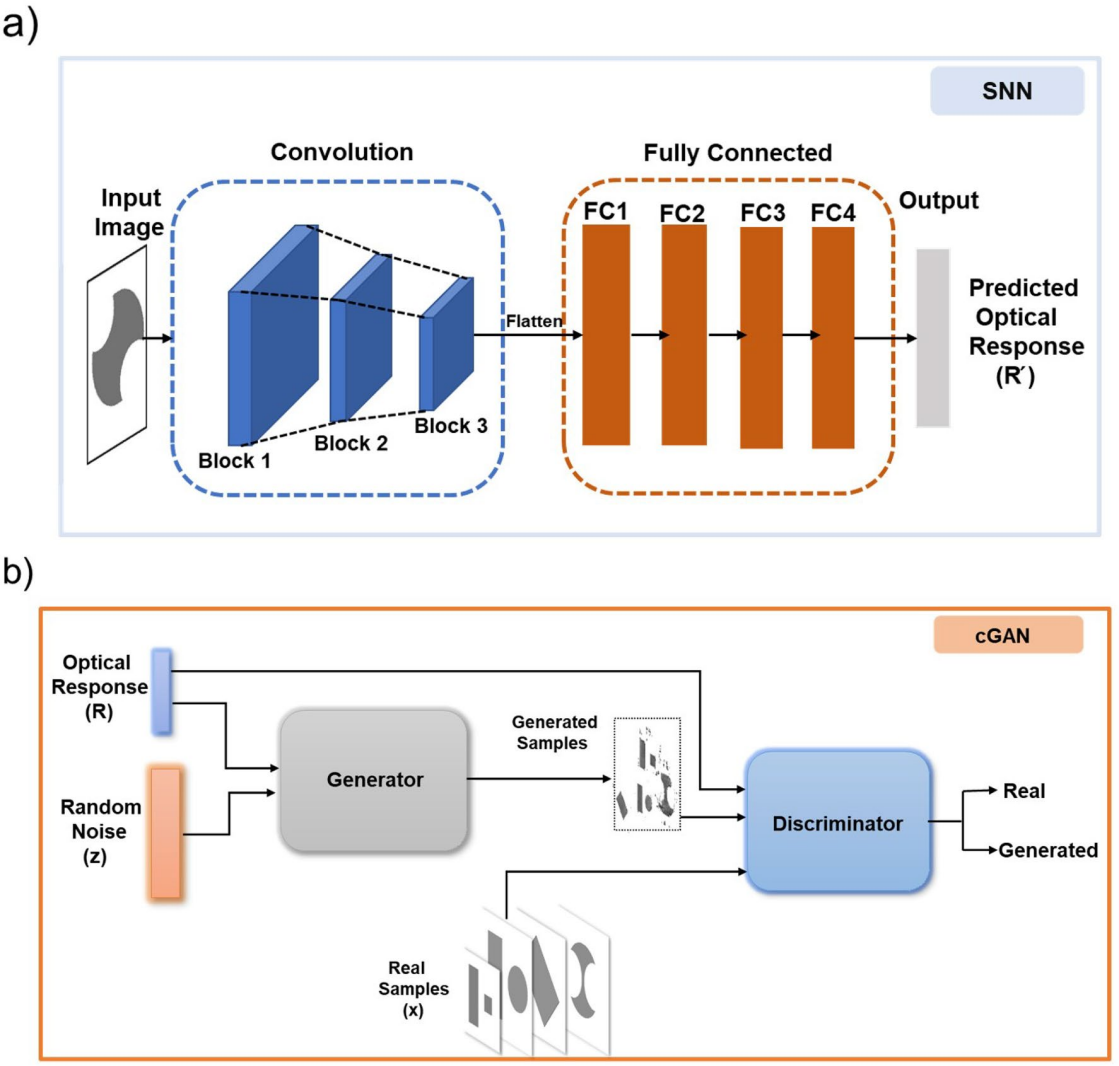
Schematic illustration of (**a**) SNN model architecture composed of CNNs and FC-NNs. It takes 2D cross sectional structural design image as input and predicts it’s optical response and (**b**) cGAN model architecture to generate structural designs. The complete architecture consists of two networks: a generator (*G*) and a discriminator (*D*). *G* accepts optical response and a random noise distribution *z* to generate structural design. *D* evaluates real structural designs from generated structural designs as real or fake.

 To predict the optical response (forward design) we use a CNN, which we call Simulation Neural Network (SNN). The SNN models the underlying non-linear relationship between the structural design of Al-nanoantennae and the corresponding optical response as a forward one-to-one mapping. Figure 2a shows the SNN model architecture which we used for forward mapping from the 2D cross sectional image (input) to the optical response (output). As shown in Fig. 2a, the convolution section (blue dashed box) of the SNN consisted of three blocks each with two convolutional layers followed by a max-pooling layer. The flattened features from the convolution section were fed to a stack of four FC layers (orange dashed box). Batch normalization was used after each layer in the tested CNN architecture. Rectified linear unit (ReLU) was used as the non-linearity in each layer except the output layer. The output layer was followed by sigmoid nonlinearity. The details of the SNN architecture are given in Table I of the Supplementary Information.

The SNN was trained using backpropagation algorithm to optimize average mean square error (MSE) between the predicted ($R^\prime$) and actual optical response (*R*) given in Equation (1) as:

1 $$\begin{aligned} L_{MSE}= \frac{1}{N}\sum _{i=1}^{N}\big (R' -R\big )^{2} \end{aligned}$$

where *N* is a mini-batch of training dataset. We used regularization methods of dropout and weight decay to avoid over-fitting. An efficiently trained SNN acts as an efficient numerical EM simulation solver that can predict an optical response for a structural design coded as an image in a few milliseconds on an inexpensive contemporary laptop computer (8GB RAM, Intel i7 four core processor).

#### The inverse design network

 We used a cGAN DL architecture^20^ to generate structural designs of meta-atom and meta-molecule as a unit of HMs for a user-defined optical response. The network produces Al-nanoantennae structural designs in the form of 2D cross sectional images for conversion efficiency as a desired optical response.

A generative adversarial network (GAN)^29^ uses a generator CNN (*G*) that takes as input a vector *z* sampled from a tractable noise distribution (e.g. Gaussian), and uses transposed convolutional layers to generate an image *G*(*z*). A second CNN called the discriminator *D* is trained to distinguish between real images and generated images. The generator is trained to fool the discriminator by learning to generate more realistic images. The discriminator is usually discarded, and the generator serves as a generative model.

A cGAN is adpated from GAN such that it takes an additional input for conditioning the output^20^ for better control over the outputs it generates (e.g. specific classes of images). The cGAN DL model is conditional with labels as additional information to guide the generation process. Here in our case, cGAN is conditional to the optical response of the structural designs. This provides details on the features present in optical response to both the generator and discriminator networks. Moreover, it ensures that the generated designs have optical response with features similar to the EM simulated dataset samples and facilitates the discriminator to classify the generated samples with information of features of optical response. The cGAN model architecture is shown in Fig. 2b. Its objective is to train the generator *G* to generate good quality Al-nanoantennae structural designs for a given input optical response (i.e., conditioned upon the desired optical response). In our cGAN model, *G* consists of five transposed convolutional layers, while *D* consists of five convolutional and two FC layers. The detailed architecture is described in Table II of the Supplementary Information. The generator takes as input a concatenation of 101 equally spaced spectral points between 400 and 800 nm of the optical response (R) and 411 Gaussian noise samples, for a total of 512 dimensional input. The choice of noise vectors for generative models^11,25,30^ does not correlate with the data samples needed for the training, since the cGAN model learns the same probabilistic distribution of EM simulation datasets (see Supplementary Information Section VII). During training, *G* learns about the conditional probability distribution of Al-nanoantennae structural design space and produces a 2D cross sectional image given an optical response as input. On the other hand, *discriminator* takes the structural design, *G*(*z*), generated from *G*, the real structural design parameter vector *x* from training dataset and desired optical response as inputs and identifies whether the design was real (actual from the training set) or fake (generated by *G*). Essentially, both *G* and *D* are trained simultaneously until *G* learns to generate nearly real structural designs to deceive *D*. During the training, *D* tries to identify fake images through minimizing the classification error while *G* tries to generate inputs so that *D* can not distinguish between real and fake images. *G* and *D* are trained to minimize and maximize the cost function respectively given in Eq. (2) as:

2 $$\begin{aligned} \min _{G}\max _{D}\ L(D,G) = E_{x\sim \text {P}_{data}(x)} \log [ D(x|R)] + E_{z\sim \text {P}_z(z)} \log [1 - D(G(z|R))] \end{aligned}$$

where *D*(*x*|*R*) represents the probability of structural design being real from training dataset for given input optical response, and *D*(*G*(*z*|*R*)) is probability of structural design generated by *G* given the input optical response. In the cGAN model, *D* is trained to maximize expectation value of *E* with $E_{x\sim \text {Pdata}(x)} \log [D(x|R)]$ for a real structural design image and $E_{z\sim \text {Pz}(z)} \log [1 - D(G(z|R))]$ for image generated by *G*. Whereas *G* is trained to give minimized expectation values to fool *D*. This adversarial training leads *G* to generate high quality 2D cross sectional image of Al-nanoantennae structural designs.

### Evaluation of DL models

For the evaluation of trained DL models, we measured the accuracy of the predicted optical response by SNN and authenticity of optical response of generated designs through cGAN, using two metrics: MSE and cosine similarity. The MSE evaluates the average error of the optical response per spectral point. We also calculated the similarity between optical responses using cosine similarity^31^, which emphasizes on matching the shape of the response instead of the magnitude, as given in Eq. (3) below:

3 $$\begin{aligned} \text {Similarity} = \cos {\theta } = \frac{\sum _{i=1}^{n} R_i R_i'}{\sqrt{\sum _{i=1}^{n} R_i^2}.\sqrt{\sum _{i=1}^{n} {R'}_i^2}} \end{aligned}$$

where *R* and $R^\prime$ are real optical response and predicted optical response respectively. The cosine similarity of 1 reflects identical characteristics between two optical responses.

Cosine similarity is better at matching the concordance of sharp variations in the optical response in the EM simulation dataset. These sharp variations include combinations of dips/peaks, oscillations and flat reflections (see Fig. 1 of Supplementary Information). Additionally, the optical responses for the training process were simulated for randomly generated structural design parameters. There may be cases where certain spectral features occur in fewer instances of the data sample thereby making it difficult for DL models to learn those features^32^. Therefore, for two optical responses as vectors in response space, the MSE measures the difference between the vectors while cosine similarity measures the similarity of features to ensure accurate prediction of optical response by SNN.

The training phase of SNN and cGAN converged in 500 and 2000 epochs, respectively using a single 4 GB Nvidia GeForce GTX 1050 GPU where each training epochs took less than one minute. The training for SNN was terminated when there was no further progress in the model’s accuracy on the validation dataset, and cGAN produced structural designs similar to real designs. The optical response predictions and generation of structural designs with the SNN and cGAN, respectively are evaluated on test samples. When MSE is minimized and cosine similarity is maximum during the training process (loss and accuracy of SNN and cGAN are included in Supplementary Information Section II), training is complete. We document the performance of SNN and cGAN on test samples to keep track of over-fitting. During training, the over-fitting occurred is minimized with batch normalization, ReLU and drop-out. We also optimize SNN with different loss functions (MSE, MAE and cosine similarity). From the study, we observe that MSE and cosine similarity as loss functions and accuracy measure, respectively, leads to much accurate prediction results on test samples (see Supplementary Information Section V).

### The cyclical generation framework

**Figure 3:**
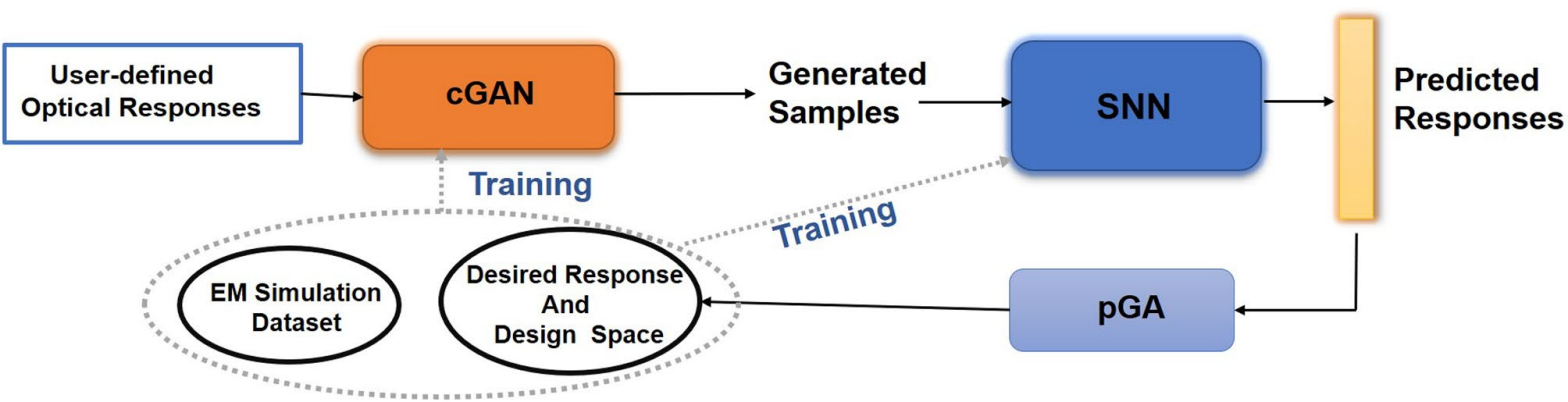
Schematic of pseudo generation framework. The framework takes as input a batch of user-defined optical responses and generates good-quality structural designs with authentic optical response using cGAN and SNN respectively, and is characterized into desired response and design space via pGA. In each cycle, the EM simulation and new generated desired response and design space data samples are used as an updated dataset for training.

Using the above mentioned models based on DL, we set up an optimization routine for Al-nanoantennae structural design for user-defined optical response. We used a pseudo genetic algorithm (pGA) to search for optimized and authentic structural designs using an objective function for designs generated from DL models. Figure 3 demonstrates the inverse design framework using both the DL models, cGAN and SNN followed by pGA. The DL models—SNN and cGAN, used in the cyclical generation framework are first pre-trained and tested for an optimal performance on unseen test samples of structural designs and their corresponding optical response.

The design procedure begins with initializing a batch of *M* user-defined desired optical response as a Gaussian mixture. The Gaussian mixture is given by:

4 $$\begin{aligned} g(\lambda )= \sum _m g_m \exp \left\{ -\frac{(\lambda - \lambda _m)^2}{2\sigma _m^2} \right\} , \end{aligned}$$

where *m*, $\lambda _m$ and $\lambda$ are the number of Gaussian, central wavelength of $m^{\text {th}}$ Gaussian and wavelength range respectively. Each sample in the batch is a variety of mixture of Gaussians introduced with random shifts in $\lambda _m$. After initialization, the batch of desired optical response ($R^d$) is input into the cGAN model to generate corresponding structural designs. Subsequently, to obtain the predicted optical response ($R^\prime$), the structural designs generated are given input into the SNN model. We now use the pseudo genetic algorithm to select the structural designs having optical response best optimized and generated in accordance with the desired optical response as input. The pGA first selects and evaluates the samples generated on the basis of the measure of their MSE and then cosine similarity.

In typical genetic algorithm technique, the problem of optimizing complex metasurface designs often involves maximization of a fitness/objective function through random mutations and cross-overs of a population of strings encoding various parameters of a design^22^. Instead, here we evaluate the optical response of generated structural designs to guide the process of design and optimization. The pGA evaluates and sorts each generated design firstly on the basis of its minimum MSE and then maximum cosine similarity score over its optical response. Therefore, we call this ‘pseudo’ genetic algorithm as we sample from the generated structural designs for its optical response deviation from desired optical response instead of sampling from the Gaussian and mutating its mean and standard deviation. We control the MSE objective function minimization by specifying a threshold value (*v*) for each batch of input optical response. This threshold incorporates maximum error propagation from both the DL models. The pGA selects $M_{\text {best}}$ desired optical response and generated structural design pairs from *M* initialized batch samples firstly for minimum MSE and then with maximum cosine similarity and sorts them in the desired response and design space. This characterized distribution of desired response and design space acts as new generation and along with EM simulation dataset (parent) is used as an updated training set. Training of cGAN and SNN again, in each cycle step, helps to move further DL models optimization and learn the latest data representation and correlation for desired response and generated designs (see Fig. 3). The new training dataset (next generation) is, after each cycle step, a combination of newly generated desired response and design space (new individuals) and dataset used in previous stage training (parents). The tuning and optimization of SNN and cGAN with the aid of pGA improves in generation of designs with optical response closely similar to the desired optical response as input. The entire DL and pGA framework performs a cyclical process of generation, simulation, selection and evaluation to create a desired response space with optimized structural designs and accurate optical responses; hence performing inverse and forward design simultaneously. In Algorithm 1, we provide an illustration of the cyclical generation framework.

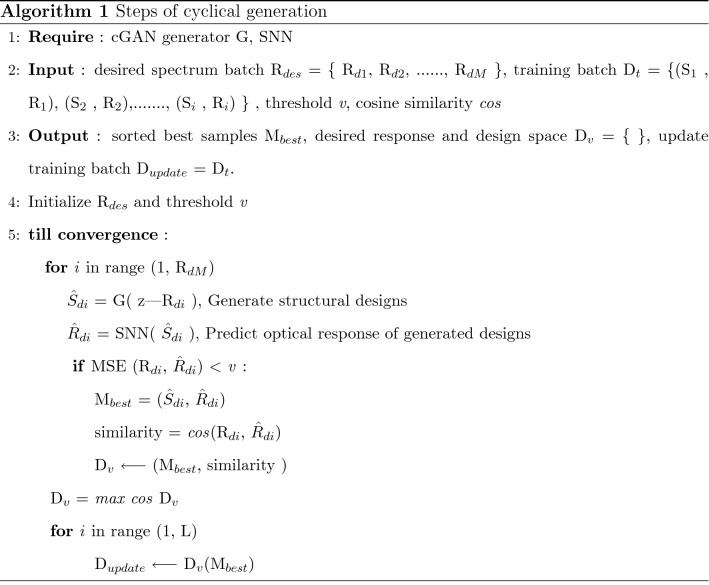



After each cyclic step, $M_{\text {best}}$ designs and response pair obtained are considered as observations and updated in training dataset for next cyclic step. In each step, the whole framework is optimized by learning updated design and response space correlations. Because the updated desired response and design space have data samples that mimic the real world user-defined responses, learning such new correlations as probabilistic data distribution could make it simpler for the cGAN and SNN to generate new designs and predict more accurate response, respectively. The cyclical generation of the desired response and design space and the updated training dataset at each step allows the associated DL models to seek for more optimal convergence and model accuracy improvement.

During the training of SNN, cGAN and the cyclical generation process, the optical responses are treated as true labels of the structural designs i.e they are the accurate optical responses that could be retrieved through numerical simulations by solving Maxwell’s equations. Hence, the most confident prediction of structural designs and optical responses by cGAN and SNN, respectively are used as an updated training dataset in cyclic step. However, we attempted training the cGAN and SNN with generated structural designs which have deviating optical response in comparison to their true EM simulation response (as the worse predictions by the network). In this scenario, we found that the MSE of SNN and cGAN increased to 0.0531 and 0.0486, respectively on test samples. Since the training of DL models is done in a supervised manner, the task becomes one of predicting the correct labels (optical response) and optimizing the network for better predictions. Hence, the cGAN and SNN are trained at each step with the good predictions of generated structural designs and accurate optical response with features similar as the simulated EM responses. Further, when the dataset is updated by incorporating such samples, it leads to a better training of network guided by pseudo genetic algorithm. The performance of cGAN and SNN improves upon including the correct predictions as the optimal designs are added to the dataset at each step.

## Results and discussion

We tested the effectiveness of the SNN and cGAN for optical response prediction and structural design generation, respectively by employing them on unseen dataset samples.

**Figure 4:**
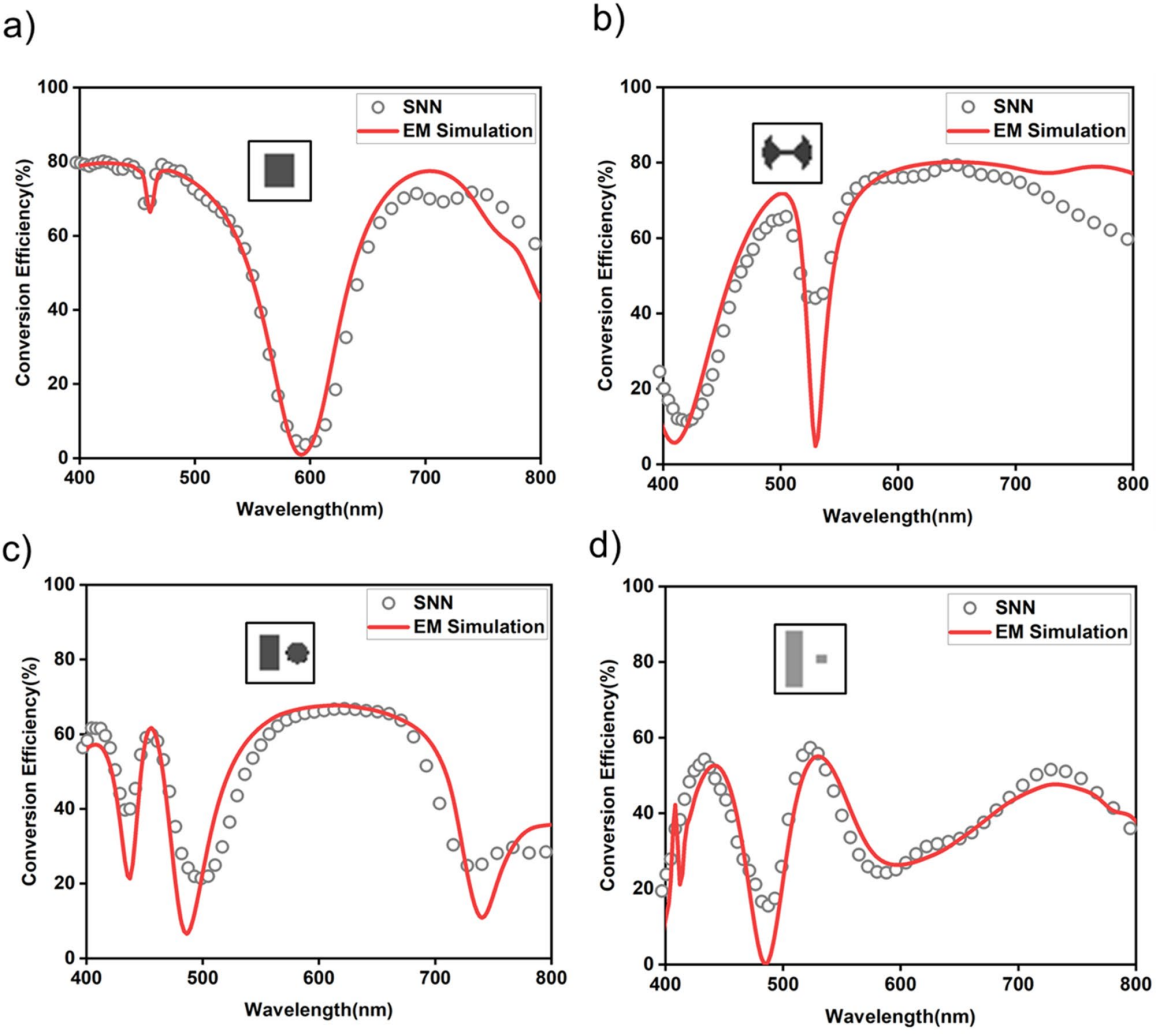
Test sample examples of SNN predictions (grey circles) with EM simulation spectrum (red curve) on structural design classes, (**a**) rectangle (**b**) double-arc (**c**) rectangle-circle pair (**d**) rectangle-square pair.

For SNN’s forward design functionality, we tested it against randomly selected Al-nanoantennae structural designs from each class to visualize SNN ’s predictability. The trained SNN predicted conversion efficiency for the unseen structural designs with an average MSE of 0.026 and a cosine similarity of 0.954. The four samples of the data shown in Fig. 4 demonstrate the accuracy of SNN on different classes of structural designs, where the real optical response from EM simulation and SNN’s predicted optical response are in good agreement. It is worth mentioning again that the prediction of optical responses using the SNN takes a only few milliseconds per sample. These results show that SNN can be used as a proxy for the time-consuming process of the forward mapping. We also note that at some sharp resonances the SNN model was not able to approximate spectra much precisely. It may be that the effect of high spectral point error at sharp resonances hence increasing the MSE of the model. When dataset have fewer data samples with such sharp resonances around certain wavelengths, it becomes particularly difficult for DL models to predict spectral points closely. Hence this error amounts to overall increase in average MSE^11,25,33–35^.

**Figure 5:**
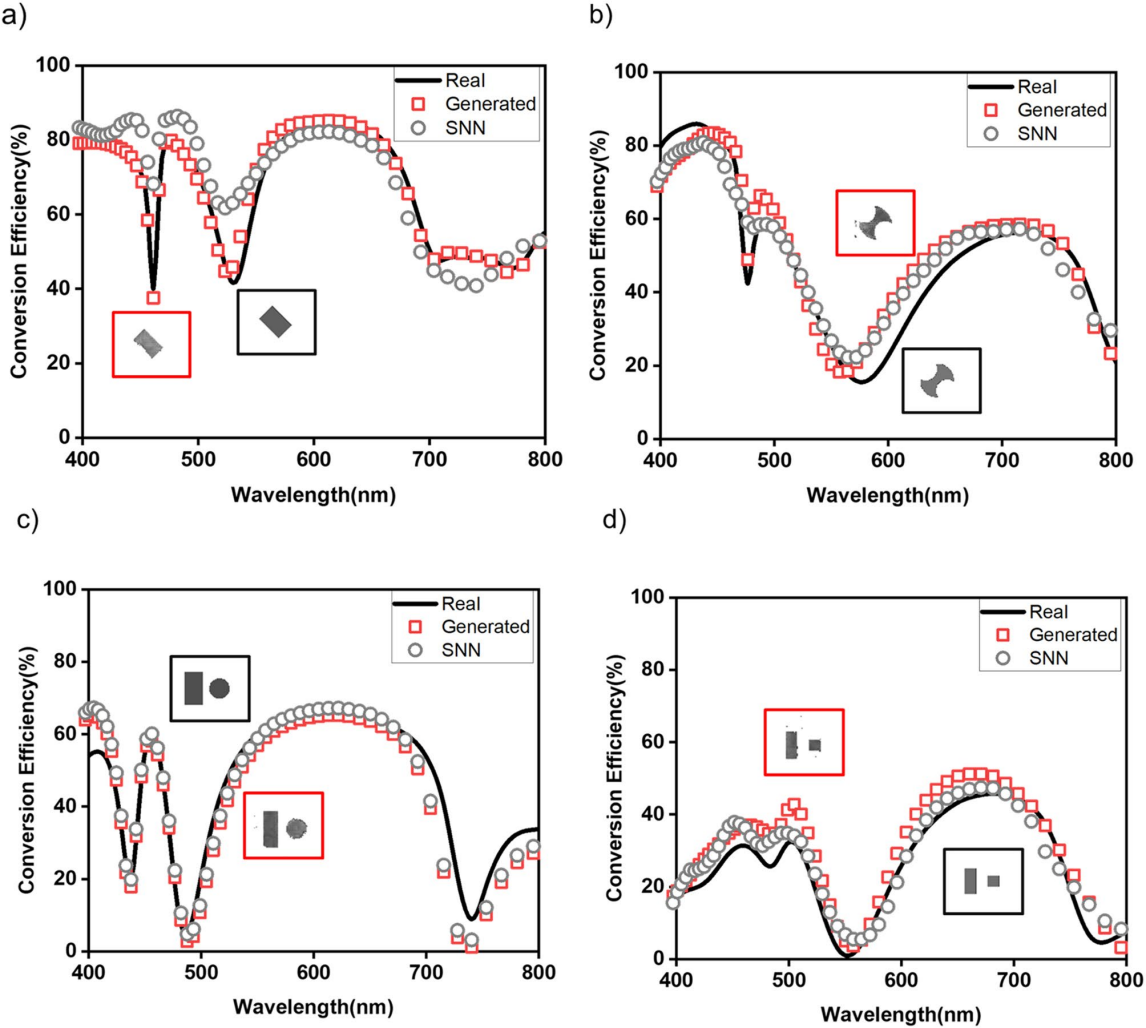
Evaluation of cGAN on test samples for each class of structural design classes, (**a**) rectangle (**b**) double-arc (**c**) rectangle-circle pair (**d**) rectangle-square pair. The EM simulation spectrum for real structural design (black curve) and generated structural design (red rectangles) along with prediction of SNN for generated structural designs (gray circles) demonstrate good quantitative agreement. Red box inset: Generated structural design, Black box inset: Real structural design.

For the inverse design process, when the training is complete, the cGAN can produce a structural design for an optical response as input in less than a second. Figure 5 shows randomly selected test results from each structural design class. The real structural designs (black box) and the corresponding structural designs generated by cGAN (red box) show good qualitative agreement. The EM simulations spectra for real structural designs (black curve) and generated structural designs (red curve) along with SNN predicted spectra for generated structural designs show excellent quantitative accuracy. For the generated and real structural designs, the average MSE and cosine similarity over the test samples is 0.011 and 0.987, respectively which highlights the potential of using the trained cGAN architecture to generate structural designs for user-defined optical response.

**Figure 6:**
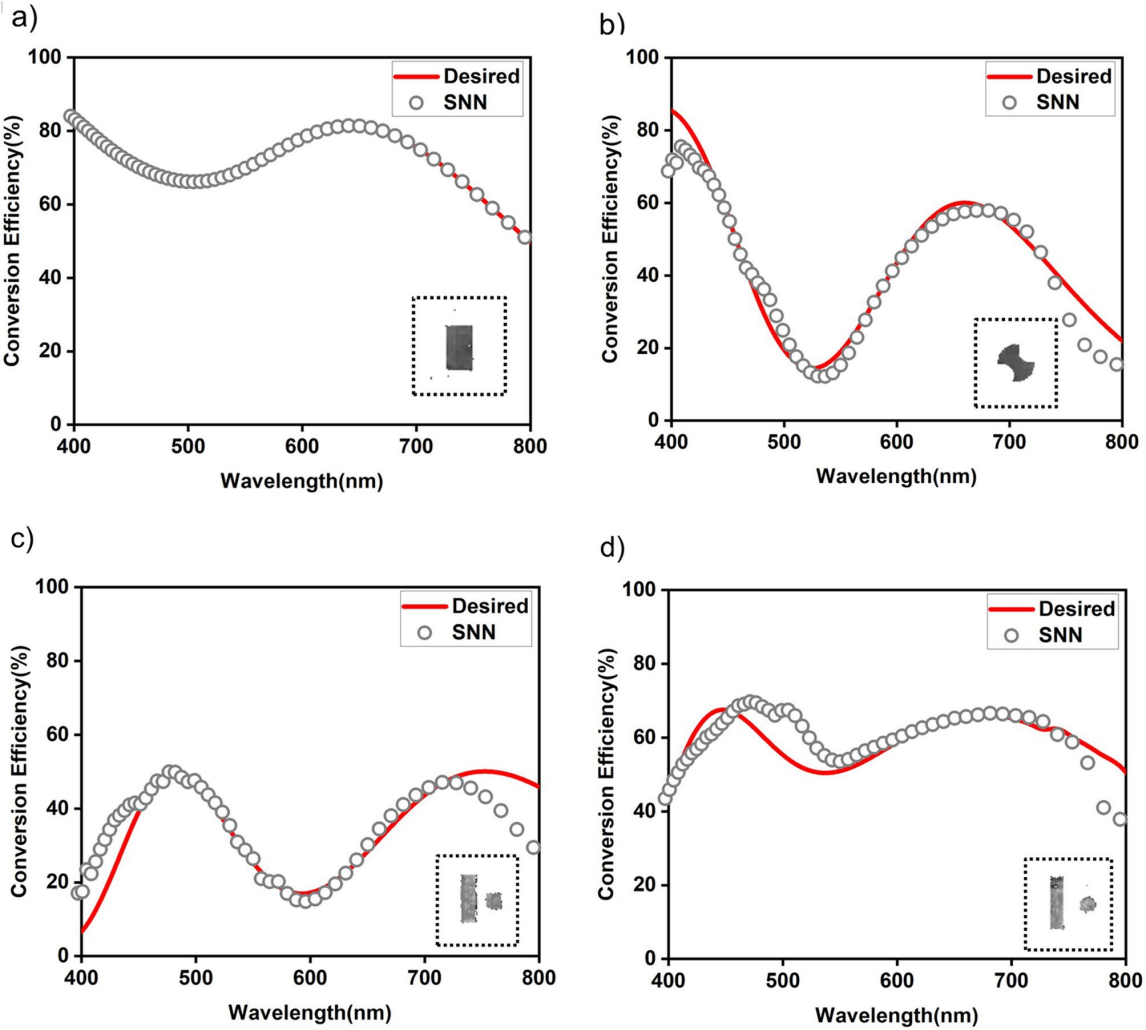
Examples of structural designs generated for the desired optical response as a Gaussian mixture. The optical response (grey circles) predicted by SNN shows good agreement with desired optical response (red curve) for generated designs(insets). (**a**) rectangle (**b**) double-arc (**c**) rectangle-square pair (**d**) rectangle-circle pair, generated design as different structural design classes.

**Figure 7:**
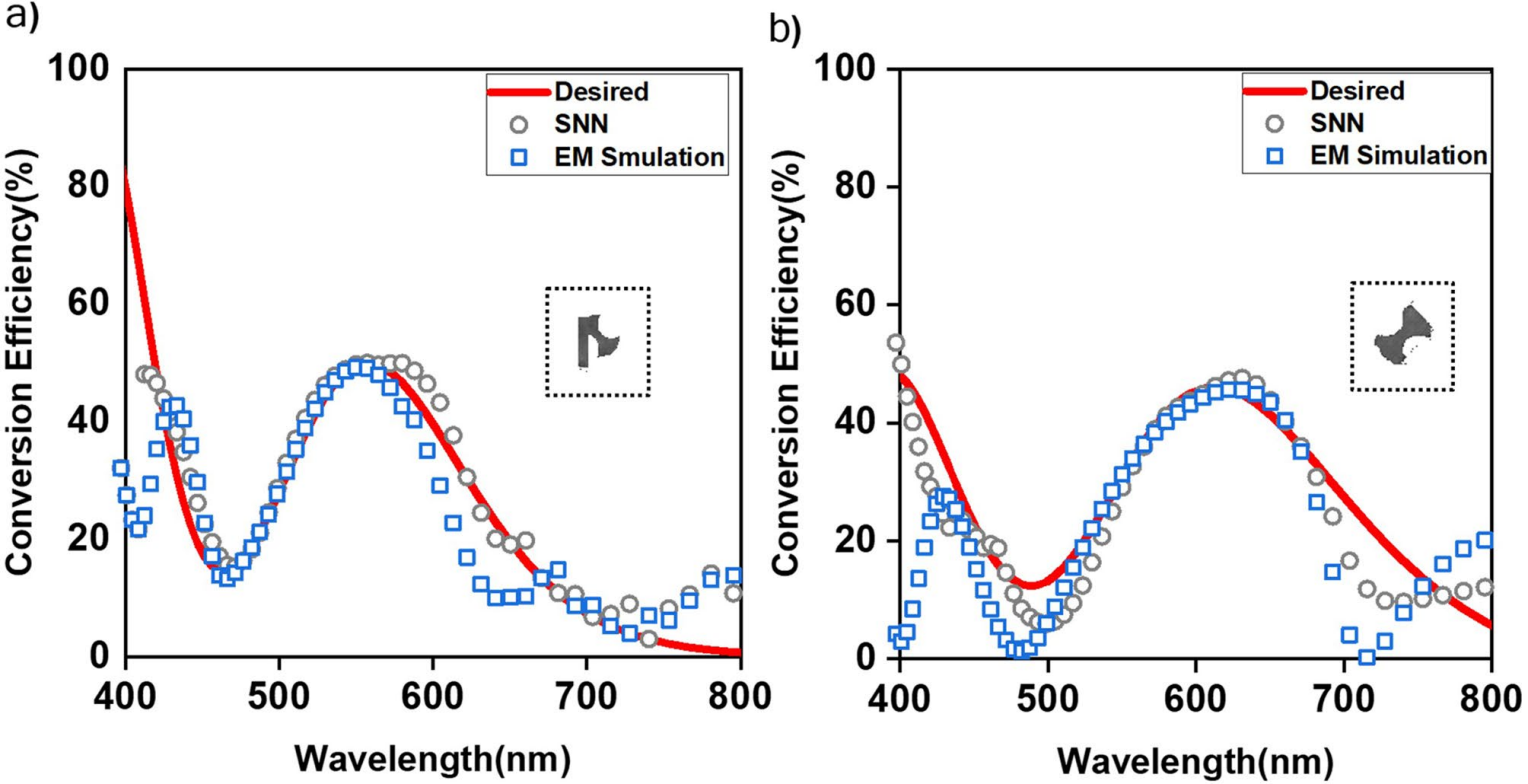
Examples of new structural designs generated for the desired optical response as a Gaussian mixture. The optical response (grey circles) predicted by SNN and EM simulation optical response (blue rectangles) shows good agreement with desired optical response (red curve) for generated designs(insets). (**a**) An axe and (**b**) dumbbell as new generated designs.

We tested the performance of our framework by inverse designing the gap-plasmon-based half-wave plate metasurface with optical responses for the desired conversion efficiency. We ran the algorithm for 5 cycles with a batch initialization of 1000 samples of desired Gaussian mixtures. The MSE threshold defined on the pGA was 0.037 which is the sum of the SNN and cGAN average MSE considering the cumulative propagation of errors from DL models. After 5 cycles, the on-demand design process results in Fig. 6 showing a few samples from the desired response and design space. Figure 6a–d show the optical response predicted by the SNN for the generated structural designs for a desired optical input response from each class. Comparing the two optical responses, it is clear that our proposed framework successfully generates designs for the desired optical response with 0.021 MSE and 0.968 cosine similarity and replicates it with only minor deviations. Our framework is robust in generating structural designs with optical response closest to the desired optical response, although there may be a possibility of design non-existence. However, after 5 cyclical frame runs, we observe the framework’s learning ability to generalize to a new structural design as shown in Fig. 7a,b, where we see new structural design as either intermediate design (an axe shape) from training designs or double arc design evolving into a dumbbell shape, respectively. We also examine the cyclical generation framework for different inputs of the desired optical response as gaussian mixtures and step functions (band filters). The structural designs generated mimic the characteristics of the desired input optical response with minimal deviations (see Supplementary Information Sections IV and VI). The updated training dataset will update the mapping between design and response space at each step. This includes correlations of the EM simulation as well as correlation of the desired optical response and the corresponding designs generated. After each cycle the DL models update their weights during each training and learn these correlations more effectively.

Importantly, with our DL methods and cyclical generation framework, the time taken for the simultaneous forward and inverse design process with optimization and evaluation is greatly reduced in comparison with the conventional EM simulation method. A comprehensive study of computing efficiency of DL models and cyclical generation framework is discussed in the Supporting Information Section III.

## Conclusion

In conclusion, our proposed framework in this paper is capable of achieving rapid and accurate inverse design of metasurfaces for a user-defined optical response with 0.021 MSE and 0.968 cosine similarity. Our framework addresses the design and optimization of metasurface as probabilistic distribution of design space and directs good-quality generation of structural design; simultaneously performing inverse and forward design using DL models. The pGA algorithm facilitates the selection of optimized generated designs to create desired optical response and design space. The generation of desired optical response and design space alleviates the need for extensive data collection through EM Maxwell’s equations solvers and drives our models towards greater generalization including the prediction of new structural designs. The generation of new designs demonstrates that our approach can be generalized to the design of other types of metasurfaces and functionalities. Additionally, this work may be expanded to generate additional data space for other DL-based optimization applications. The results of our deep learning models indicate that this type of framework is a powerful tool to reduce the cost of computation and optimize nanophotonic design efficiency while exploring new designs.

## Supplementary information

Supplementary information

## Data Availability

The datasets generated during and/or analysed during the current study are available from the corresponding author on reasonable request.
