# Supplementary material for: A cyclical deep learning based framework for simultaneous inverse and forward design of nanophotonic metasurfaces
Source: Sci Rep. 2020 Nov 10;10:19427. doi: 10.1038/s41598-020-76400-y (PMC7656263; doi:10.1038/s41598-020-76400-y)
Supplement: Supplementary file 1 — Supplementary information [file 41598_2020_76400_MOESM1_ESM.pdf]

A Cyclical Deep Learning Based Framework For Simultaneous Inverse and Forward Design of Nanophotonic Metasurfaces

Abhishek Mall,[1] Abhijeet Patil,[2] Amit Sethi,[2,*] and Anshuman Kumar[1,†]

[1]*Department of Physics, Indian Institute of Technology – Bombay, Mumbai - 400076, India*

[2]*Department of Electrical Engineering,*
Indian Institute of Technology – Bombay, Mumbai - 400076, India

[*] asethi@iitb.ac.in

[†] anshuman.kumar@iitb.ac.in

SUPPLEMENTARY INFORMATION

I. Variation in optical response of training samples

Fig. 1 depicts randomly selected data samples from the dataset used for training DL models. The optical responses corresponding to each data sample include sharply varying features such as dips / peak (red arrow), oscillations (orange arrow), and flat (blue arrow) reflections. The appearance in every optical response of a combination of these features induces difficulty in mapping structural design to optical response. The dataset with fewer data samples with these variations could result in deviation in predicting optical response when a NN is being trained on this dataset. Those data samples may act as outliers with spectral response variation. The MSE loss function penalizes outliers in dataset while cosine similarity ensures precise feature prediction.

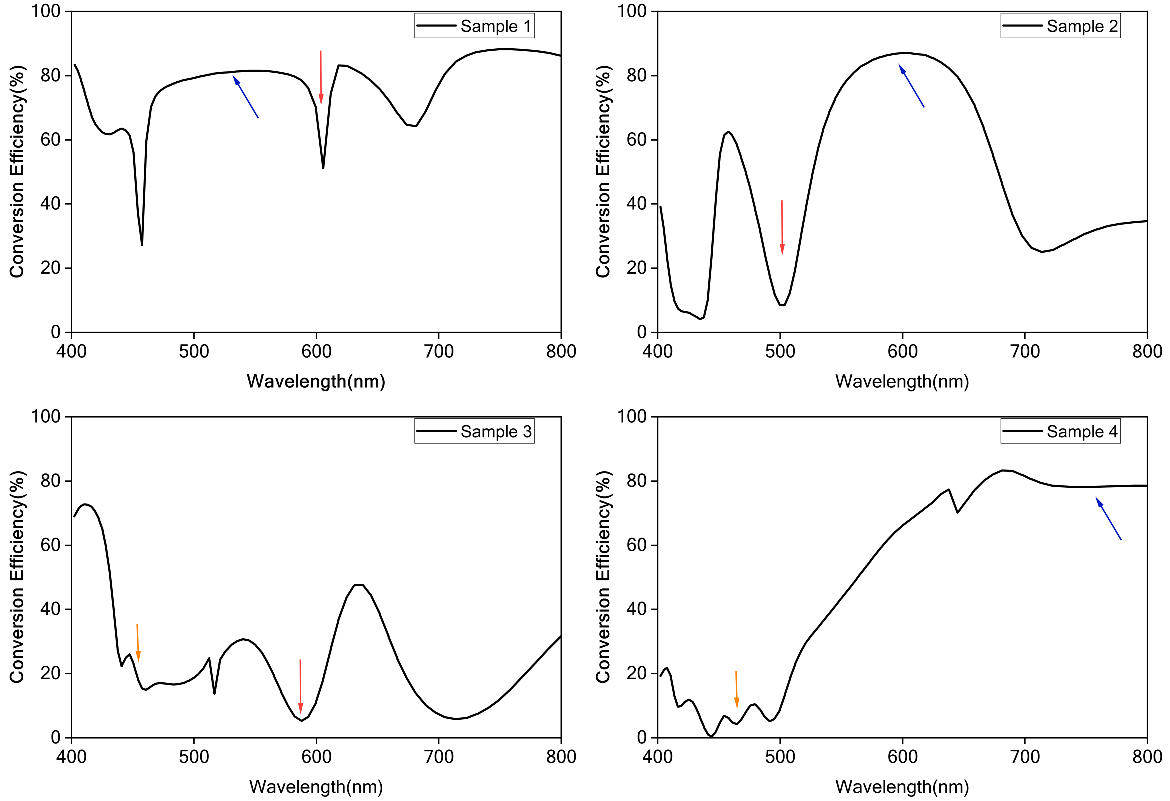

Figure 1 : Data samples of optical response from EM simulation generated training dataset

II. Loss and accuracy of DL models : SNN and cGAN

SNN Training: The SNN training is completed after 500 epochs, when on test data samples the average MSE and cosine similarity for predicting optical responses stabilizes at 0.026 and 0.954 respectively. The SNN training took 4 hours (see our machine specifications on page 8) before MSE and cosine similarity were not improving further for later epochs. During SNN training the loss and similarity were reported (see Figure 2). During training, the over-fitting occurred is minimized with batch normalization, ReLU and drop-out. Using these regularizations, the over-fitting has been reduced such that the MSE is minimum and the cosine similarity is maximum on the test samples, indicating that the SNN has converged and the training can be terminated.

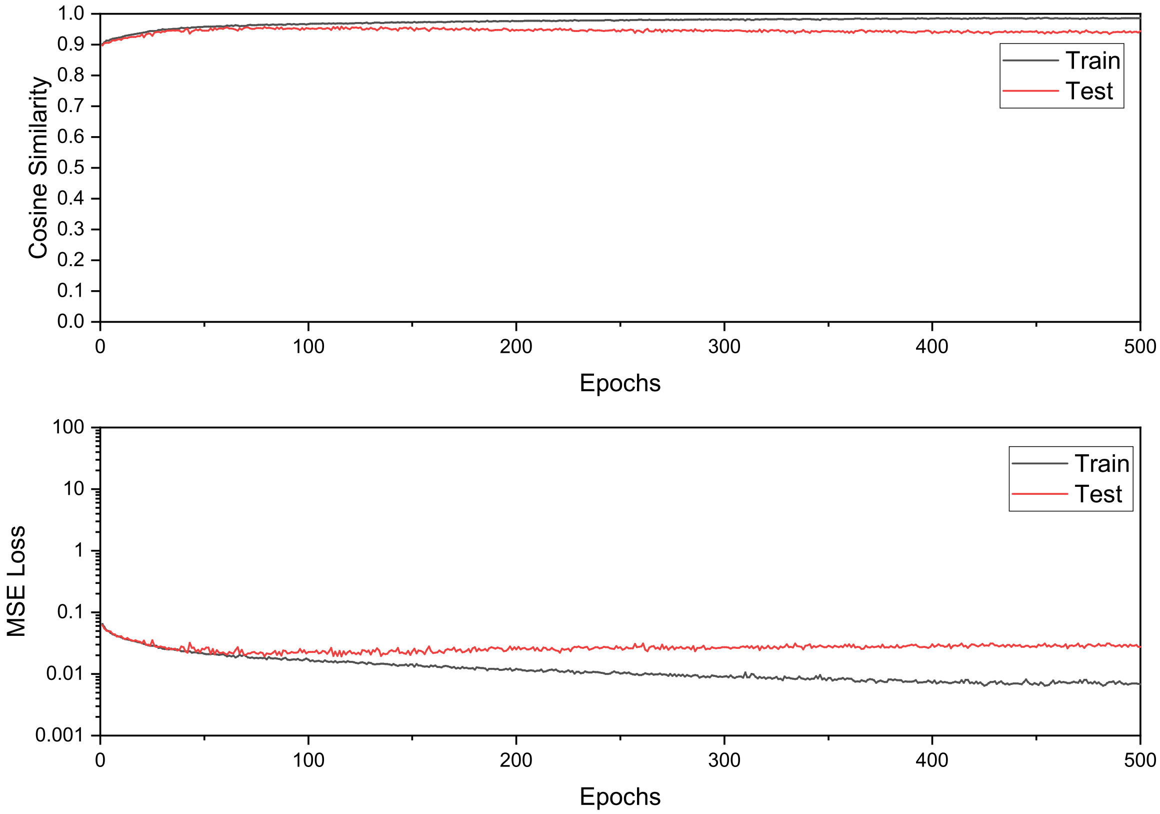

Figure 2 : Training and test curves of MSE loss and cosine similarity for SNN.

cGAN Training : The discriminator and generator loss for cGAN is shown in Figure 4 until the training was terminated at 2000 epochs. We demonstrate the generating ability of cGAN through it's probabilistic representation learning after certain epochs as shown in Figure 3. The evolution of generation ability for structural designs after certain epochs shows that the generator produces designs which are closely similar to real designs from training data. Once the training is over, we test the cGAN network on test samples and evaluate the optical response of structural design produced by means of EM simulation and SNN. In main text Figure 5, we show such test samples to evaluate the performance of cGAN. We observe that the structural designs generated have optical response in agreement with EM simulation and SNN predictions.

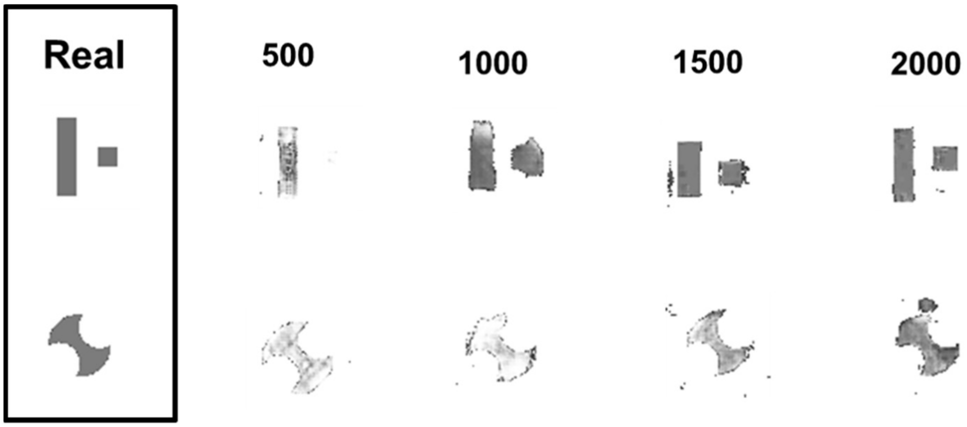

Figure 3 : Evolution of generated structural designs by cGAN after certain epochs. After 2000 epochs, the generated design patterns are very similar to real designs.

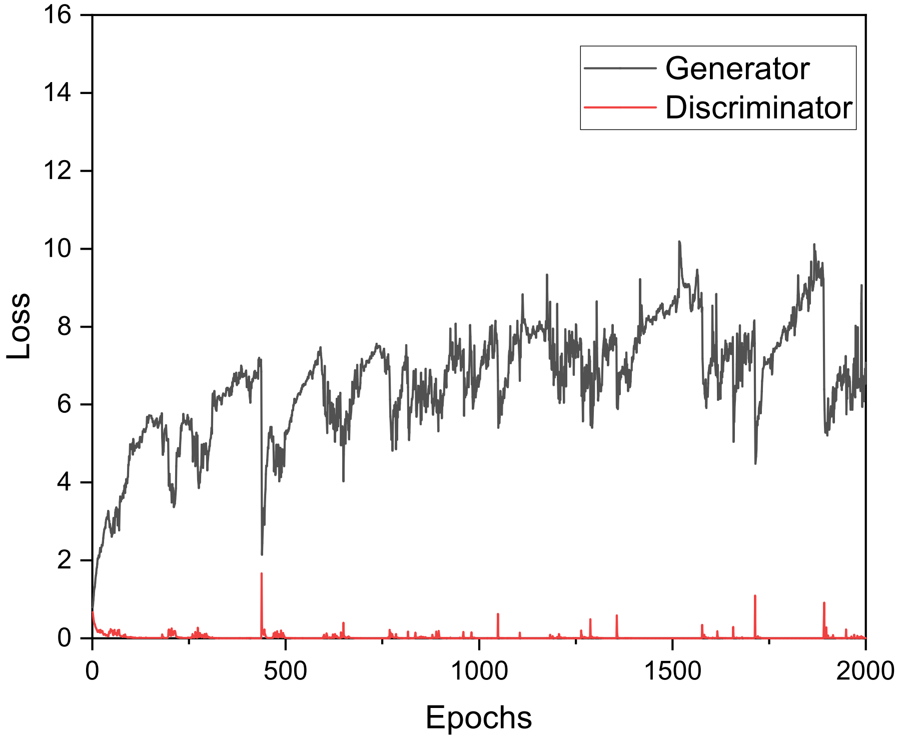

Figure 4 : cGAN: Loss curves for generator and discriminator network.

TABLE I: **Detailed information of simulation neural network(SNN) model architecture and parameters**

**Convolutional Layers**			
**Layer type**	**Block 1**	**Block 2**	**Block 3**
**Conv2d**	[1, 32, 3, 1]	[64, 128, 3, 1]	[128, 256, 3, 1]
**Batch Norm2d**	32	128	256
**ReLU**			
**Conv2d**	[32, 64, 3, 1]	[128, 128, 3, 1]	[256, 256, 3, 1]
**Batch Norm2d**	64	128	256
**ReLU**			
**Maxpool2d**	(2,2)	(2,2)	(2,2)
**Dropout2d**	p = 0.5	p = 0.5	p = 0.5

**Fully connected Layers**				
**Layer type**	**FC 1**	**FC 2**	**FC 3**	**FC4**
**Linear**	4096	2048	1024	512
**Dropout**	-	p = 0.5	p = 0.5	p = 0.5
**ReLU**	-			

Note: In the tables above, **Conv2d**, **Batch Norm2d**, **ReLU**, **Maxpool2d**, **Dropout2d**, and **FC** represent convolutional, batch normalization, rectified linear, max pooling, dropout, and fully connected layers, respectively, while the square brackets capture [input channels, output channels, kernel size, padding] of convolutional layers, and parentheses capture (kernel size, stride) of the max pool layer.

TABLE II: **Network structure and parameters for cGAN model**

**Layer type**	**Generator**	**Layer type**	**Discriminator**
**ConvTranspose2d**	[512, 512, 4, 1, 0]	**Conv2d**	[1, 64, 4, 2, 1]
**Batch Norm2d**	512	**Batch Norm2d**	64
**ReLU**		**LeakyReLU(0.2)**	
**ConvTranspose2d**	[512, 256, 4, 2, 1]	**Conv2d**	[64, 128, 4, 2, 1]
**Batch Norm2d**	256	**Batch Norm2d**	128
**ReLU**		**LeakyReLU(0.2)**	
**ConvTranspose2d**	[256, 128, 4, 2, 1]	**Conv2d**	[128, 256, 4, 2, 1]
**Batch Norm2d**	128	**Batch Norm2d**	256
**ReLU**		**LeakyReLU(0.2)**	
**ConvTranspose2d**	[128, 64, 4, 2, 1]	**Conv2d**	[256, 512, 4, 2, 1]
**Batch Norm2d**	64	**Batch Norm2d**	512
**ReLU**		**LeakyReLU(0.2)**	
**ConvTranspose2d**	[64, 1, 4, 2, 1]	**Conv2d**	[512, 100, 4, 2, 1]
**Tanh**		**Flatten**	concatenate
			101×1 spectrum
		**FC**	512
		**Batch Norm1d**	512
		**LeakyReLU(0.2)**	
		**FC**	1
		**Sigmoid**	

TABLE III: **Hyper-parameters for training of cGAN and SNN**

**Hyper-Parameters**	**Generator**	**Discriminator**	**SNN**
**Batch Size**	64	64	64
**Learning Rate**	2e-4	2e-4	3e-4
**Optimizer**	Adam	Adam	Adam

TABLE IV: **Time comparison**

**Task**	**Duration**
Dataset generation	50 hours
Training of SNN	4 hours
Training of cGAN	8.5 hours
Optical response prediction (Forward design)	**SNN:** 4.2ms, **COMSOL:** 1min 45s
Structural design generation (Inverse Design)	**cGAN:** 5.5ms
Cyclical generation framework (1000 designs)	11s
Cyclical generation framework (single design)	11ms

III. Computing Efficiency : DL models and Cyclical Generation Framework vs EM Simulation

We evaluated the computing efficiency of DL models and the cyclical generation framework by documenting the time taken to carry out forward design and inverse design on test data samples. For a fair comparison, the computation time was assessed using the same contemporary laptop computer. The laptop hardware consists of a single 4 GB Nvidia GeForce GTX 1050 GPU, 8 GB RAM, and Intel i7 four core processor. We measured the time-taken for all tasks performed using DL models and cyclical generation framework. All EM simulations were performed using FEM-based electromagnetic simulation software: COMSOL MULTIPHYSICS with Livelink for MATLAB. In Table IV, we observe that DL models and framework once optimized are more efficient than conventional EM simulation method. Importantly, the time taken for the simultaneous forward and inverse design with optimization process using cyclical generation framework is very fast, indicating that the design and optimization of metasurfaces is achieved on low computation cost within very small time scales.

IV. Cyclical Generation Framework : Example samples for user-defined optical response as Gaussian mixture.

We show more examples (see Figure 5) of generated structural designs from the cyclical generation framework for different desired optical response as Gaussian mixtures. In each example, the inset figure is the corresponding generated structural design.

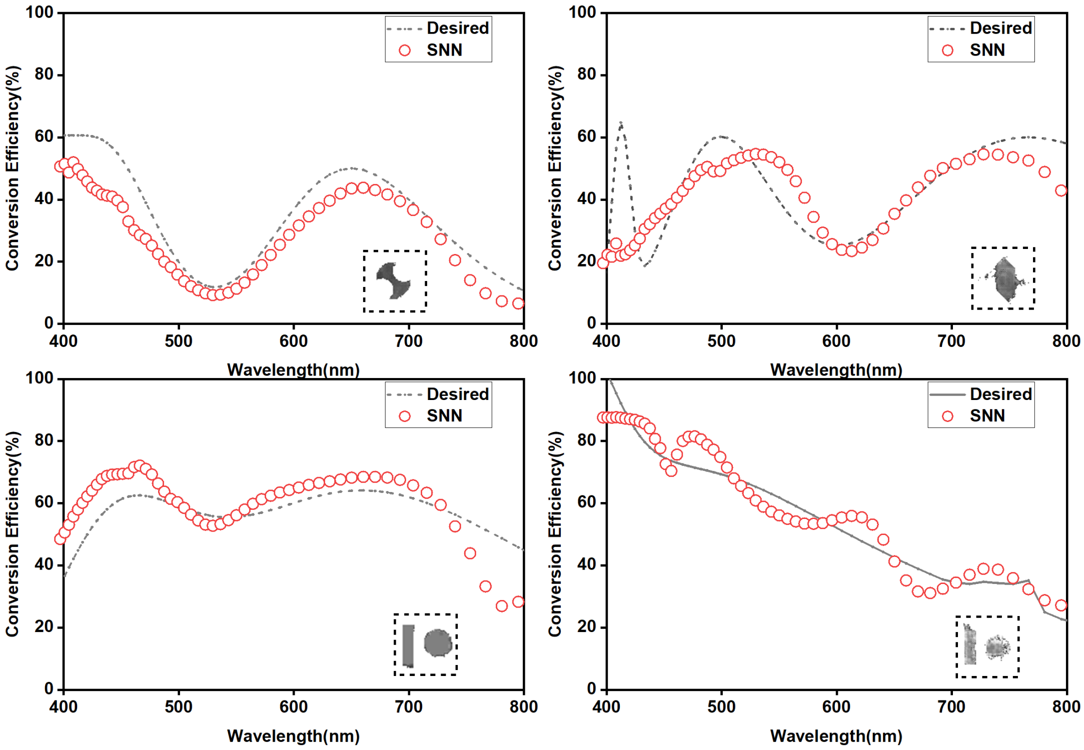

Figure 5 : Examples of structural designs generated for the desired optical response as a Gaussian mixture.

V. Optimization of SNN with different loss functions.

In order to understand the choice of loss function for SNN optimization and accuracy calculation for optical response prediction, we implement SNN training with loss functions such as MSE, MAE and cosine similarity, respectively. In Table V, we optimize SNN with different loss functions and evaluate the optical response prediction with different accuracy measures. Comparing results for different optimizations, we find that MSE as a loss function and cosine similarity as an accuracy measure leads to a better evaluation for accurate optical response prediction. Moreover, Figure 6 shows that when cosine similarity is used as a loss function for SNN's training and optimization, the SNN is able to learn the features of the optical response. However, each spectral point has a high MSE leading to a large average MSE of 0.066 on the test samples.

TABLE V: **Loss functions for optimization of SNN**

**Loss function**	**Accuracy Measure**
**MSE**: 0.026	**Cosine Similarity**: 0.954
**MAE**: 0.17	**Cosine Similarity** : 0.935
**Cosine Similarity**: 0.946	**MSE**: 0.066

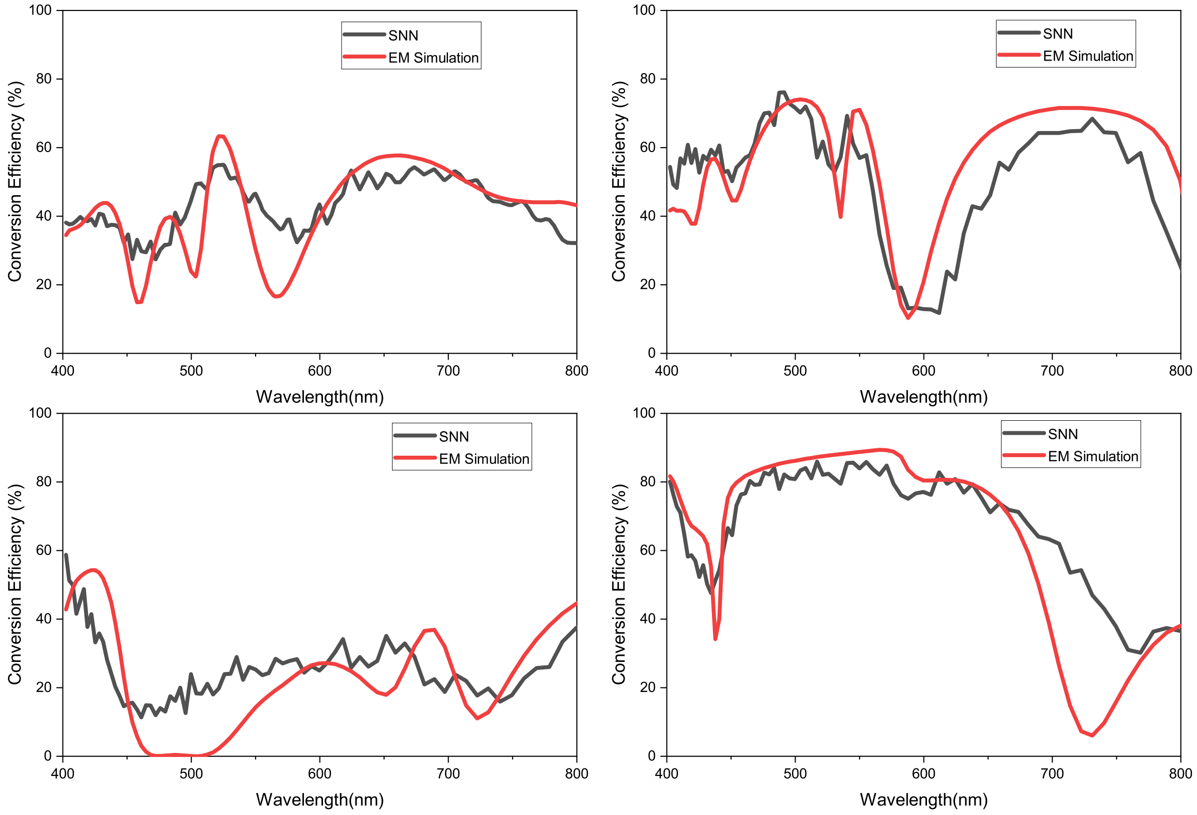

Figure 6 : Test sample examples of SNN predictions for cosine similarity as loss function and MSE as accuracy measure.

VI. Cyclical Generation Framework : Example samples for user-defined optical response as step functions (band filters).

The cyclical generation framework is capable of generating authentic structural designs, most likely from the learned design class, such that the optical response of the generated structural designs mimics the features of the desired input optical response with minimal deviations. In Figure 7, we show examples of generated structural designs patterns for different user defined (band filter) optical response. The generated structural designs (inset) closely capture the features of desired optical response.

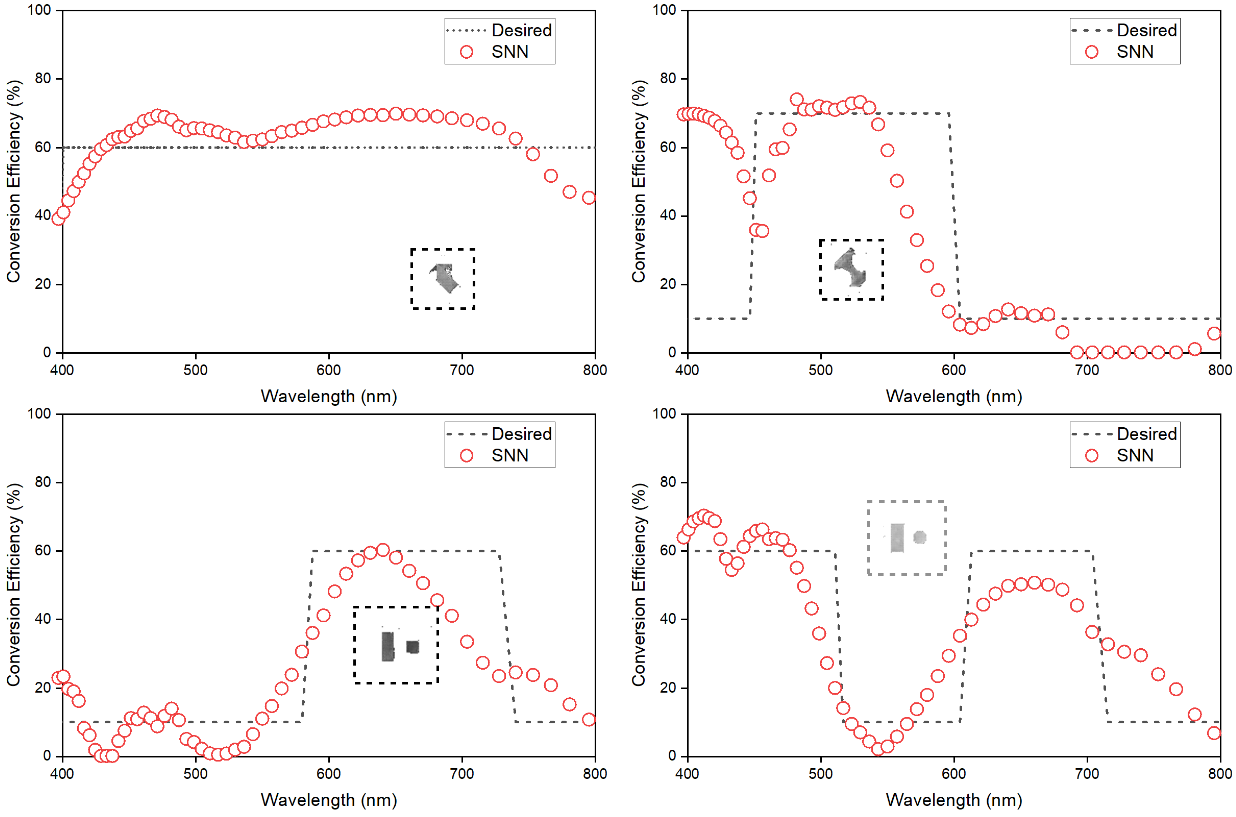

Figure 7 : Examples of structural designs generated for the desired optical response as step functions (band filters).

TABLE VI: **Training of cGAN with different dimension of noise vectors**

**Input Vector to G**	**Noise Vector**	**Performance**
256	155	MSE : 0.010, Cosine Similarity : 0.991
512	411	MSE : 0.011, Cosine Similarity : 0.987
1024	923	MSE : 0.0115 , Cosine Similarity : 0.989

VII. Different noise vector dimension input to cGAN.

We perform the training of cGAN with different dimension of input noise vector to understand the relation between the noise vector space and the dataset used for training. Table VI shows the performance of the cGAN on test samples as a measure of MSE and cosine similarity. We observe that for different dimension of input noise vectors, the performance of cGAN remains fairly same in all the cases. The choice of noise vectors for generative models [1–3] does not correlate with the data samples needed for the training, since the cGAN model learns the same probabilistic distribution of EM simulation datasets for each case listed in Table VI.

[1] J. A. Hodge, K. V. Mishra, and A. I. Zaghloul, "Multi-discriminator distributed generative model for multi-layer rf metasurface discovery," in 2019 IEEE Global Conference on Signal and Information Processing (GlobalSIP), pp. 1–5, IEEE, 2019.

[2] Z. Liu, D. Zhu, S. P. Rodrigues, K.-T. Lee, and W. Cai, "Generative model for the inverse design of metasurfaces," Nano letters, vol. 18, no. 10, pp. 6571–6576, 2018.

[3] S. So and J. Rho, "Designing nanophotonic structures using conditional deep convolutional generative adversarial networks," Nanophotonics, vol. 8, no. 7, pp. 1255–1261, 2019.
